# Receptor for Advanced Glycation End-Products Signaling Interferes with the Vascular Smooth Muscle Cell Contractile Phenotype and Function

**DOI:** 10.1371/journal.pone.0128881

**Published:** 2015-08-06

**Authors:** Elie Simard, Thomas Söllradl, Jean-Sébastien Maltais, Julie Boucher, Pédro D’Orléans-Juste, Michel Grandbois

**Affiliations:** 1 Département de Pharmacologie et physiologie, Faculté de Médecine et des Sciences de la Santé de l’Université de Sherbrooke, Sherbrooke, Canada; 2 Institut de Pharmacologie de Sherbrooke, Université de Sherbrooke, Sherbrooke, Canada; 3 Chaire de Recherche Canadienne en Nanopharmacologie et Microscopie à Force Atomique, Faculté de Médecine et des Sciences de la Santé de l’Université de Sherbrooke, Sherbrooke, Canada; Pennsylvania State Hershey College of Medicine, UNITED STATES

## Abstract

Increased blood glucose concentrations promote reactions between glucose and proteins to form advanced glycation end-products (AGE). Circulating AGE in the blood plasma can activate the receptor for advanced end-products (RAGE), which is present on both endothelial and vascular smooth muscle cells (VSMC). RAGE exhibits a complex signaling that involves small G-proteins and mitogen activated protein kinases (MAPK), which lead to increased nuclear factor kappa B (NF-κB) activity. While RAGE signaling has been previously addressed in endothelial cells, little is known regarding its impact on the function of VSMC. Therefore, we hypothesized that RAGE signaling leads to alterations in the mechanical and functional properties of VSMC, which could contribute to complications associated with diabetes. We demonstrated that RAGE is expressed and functional in the A7r5 VSMC model, and its activation by AGE significantly increased NF-κB activity, which is known to interfere with the contractile phenotype of VSMC. The protein levels of the contraction-related transcription factor myocardin were also decreased by RAGE activation with a concomitant decrease in the mRNA and protein levels of transgelin (SM-22α), a regulator of VSMC contraction. Interestingly, we demonstrated that RAGE activation increased the overall cell rigidity, an effect that can be related to an increase in myosin activity. Finally, although RAGE stimulation amplified calcium signaling and slightly myosin activity in VSMC challenged with vasopressin, their contractile capacity was negatively affected. Overall, RAGE activation in VSMC could represent a keystone in the development of vascular diseases associated with diabetes by interfering with the contractile phenotype of VSMC through the modification of their mechanical and functional properties.

## Introduction

Chronic hyperglycemia leads to the formation of sugar-derived adducts referred to as advanced glycation end-products (AGE) [1]. AGE result from a slow chemical reaction between sugars and amine groups present in proteins, lipids or DNA [2]. Glycation of plasma proteins, such as albumin and hemoglobin, is universally used as a marker of chronic hyperglycemia in diabetic patients [3]. During AGE formation, primary amines in proteins slowly react with glucose, its autoxidation products or other glycolysis intermediates, such as glyoxal or methylglyoxal [4]. A variety of AGE structures have been identified, such as carboxymethyl-lysine (CML), carboxyethyl-lysine, pyrralyine, pentoside, imidazolone, and pyrimidine [5]. An important aspect of AGE formation is the activation of the receptor for advanced glycation end-products (RAGE) [6]. It has been previously suggested that sustained RAGE activation via AGE present in the blood plasma could lead to an aberrant activation of multiple signaling pathways, such as the small G protein RhoA, Cdc42, Rac and Ras [7], protein members of the mitogen activated protein kinase (MAPK) family extracellular signal-regulated kinases 1/2 (ERK1/2), c-Jun N-terminal kinase (JNK) and P38 [8] or Janus kinase/signal transducer and activator of transcription (JAK/STAT) and phosphatidylinositol-4,5-bisphosphate 3-kinase/protein kinase B (PI3K/AKT) kinases [9]. Interestingly, several pathways are involved in the activation of nuclear factor kappa B (NF-κB), which participates in the regulation of more than 150 genes related to inflammation, cell proliferation, immune system modulation or apoptosis [10]. RAGE activation also causes an important oxidative stress via an increased production of reactive oxygen species that further reinforce NF-κB activation [11]. Therefore, it is suggested that RAGE-dependent NF-κB activation in vascular cells could be an important contributor to vascular dysfunction. Interestingly, RAGE expression is also positively regulated by RAGE-dependent NF-κB activation [12]. Through this positive feedback mechanism, RAGE activation may exacerbate the chronic inflammation commonly associated with diabetes.

Vascular smooth muscle cells (VSMC) are the mechanically active cell layer in the vascular system, and they are directly responsible for the regulation of blood pressure and blood flow distribution [13]. A deregulation of the vascular smooth muscle cell phenotype and contractile function can lead to hypertension and complications, such as cardiac hypertrophy and failure, renal dysfunction and cerebrovascular diseases [14]. VSMC are not terminally differentiated cells. While their phenotype is predominately contractile, mechanical, hormonal and environmental stimuli, such as alterations in the extracellular matrix, can lead to a switch toward a synthetic and proliferative phenotype. This phenotypic plasticity allows VSMC to fulfill divergent roles, such as the active regulation of vascular tone and arterial remodeling, repair and growth [15].

A major regulator of the contractile phenotype of VSMC is the serum response factor (SRF), which plays a pivotal role in the regulation of many contractile associated genes, such as smooth muscle alpha-actin (SM-α-actin), smooth muscle myosin heavy chains (SM-MHC) and transgelin (SM-22α), and thereby maintains VSMC contractile function [16]. Myocardin (MyoC) and myocardin related transcription factor (MRTF) are two transcriptional coactivators which, together with SRF, are responsible for the maintenance of VSMC in a differentiated contractile phenotype. Furthermore, small GTPases, such as RhoA, also play an essential role in the regulation of the VSMC contractile phenotype by increasing actin-dependent nuclear translocation of MRTF. Conversely, the activation of the MAPK ERK 1/2 pathway or transcription factor NF-kB, through the inhibition of MyoC or MRTF association with SRF, leads to a phenotype switch via down-regulation of the expression of proteins involved in the contractile phenotype [17].

Several lines of evidence suggest a role for RAGE in the regulation of genes involved in the contractile phenotype of VSMC. Little is known regarding the impact of RAGE activation on the mechanical properties of VSMC; thus, we aimed to delineate the impact of its activation on the phenotype of VSMC and the downstream consequences of this signaling interplay on their functional mechanical activity. Here, we demonstrate that VSMC express the AGE receptor RAGE and are sensitive to various AGE derived from human serum albumin (HSA). Using AGE-HSA, as well as the specific protein adduct HSA-CML, we demonstrate that RAGE activation interferes with the VSMC phenotype via modification of the expression of important contractile marker proteins and regulators associated with their contractile phenotype. Finally, we demonstrate that RAGE activation decreases the ability of VSMC to respond to vasopressin (AVP), which is a vasoactive hormone known to induce vascular smooth muscle cell contraction. Taken together, our results support the hypothesis that RAGE activation can modify vascular homeostasis via the induction of VSMC de-differentiation and loss of contractile function.

## Materials and Methods

### Reagents

Vasopressin was purchased from American Peptide Company (66-0-03). Phosphate buffered saline (PBS; 311-425-CL) and Hepes (330-050-EL) were purchased from Wisent. Fura-2/AM was obtained from Life Technologies (F-1201). Tris(hydroxymethyl)aminomethane (T395-1), NaCl (S671), KCl_2_ (P217) and CaCl_2_ (C70) were obtained from Fisher. Glycine (G7126), Glucose (G8270) and EGTA (E0396) were purchased from Sigma. MgSO_4_ (AC-5568) was ordered from Anachemia.

### Cell culture

Rat aortic/thoracic vascular smooth muscle cells (A7r5) (CRL-1444; ATCC) were maintained in minimum essential Dulbecco’s modification Eagle’s medium (DMEM) (319–005; Wisent), which contained 10% FBS, 0.6 IU/ml penicillin (450-201-EL; WISENT), 600 μg/ml streptomycin (450-201-EL; WISENT) and non-essential amino acids (NEAA) (312–012; Wisent). Cells were grown on 10 cm petri dishes (353003; Corning), unless specified otherwise, at 37°C in a humidified atmosphere with 5% CO2. The cells were passed twice per week until confluence was attained and starved 24 hours prior to experiments because FBS contains AGE harboring CML adducts (Figure A in S1 File).

### Glycation of albumin

To investigate RAGE activation from AGE, AGE derived from the long term incubation of albumin with glucose is widely used. Here, two types of glycated HSA were synthetized: one HSA contained a wide variety of AGE referred to as AGE-HSA, and one HSA contained only CML adducts referred to as CML-HSA. Glycated recombinant human serum albumin (HSA) (A9731; Sigma) was prepared as previously described [18]. Briefly, AGE-HSA was produced via incubation of 50 mg/ml HSA with 30 mM D-Glucose PBS at 37°C in the dark for 6 weeks. CML-HSA was produced via incubation of 50 mg/ml HSA in 0.1 M phosphate buffer, pH 7.4, with 45 mM Gloxylic acid (128465; Sigma) and 150 mM sodium cyanoborohydride (156159; Sigma) at 37°C in the dark for 24 hours. Synthesized AGE were also dialyzed 48 hours at 4°C to remove excess reagents and filtered on 0.2 μm (83.1826.001; Sarstedt); the final protein concentrations were also determined using a BCA assay [19]. All AGE were produced using sterile materials in sterile conditions.

### Free primary amine assay

Trinitrobenzene sulfonic acid (TNBSA) readily reacts with the primary amino groups of amino acids in aqueous solution at pH 8 to form yellow adducts, whereas no colored derivatives are formed with the secondary amino acids proline and hydroxyproline. TNBSA has been used as a hydrophilic modifying reagent for the detection of solvent-exposed primary amines in samples that contain amino acids, peptides or proteins [20]. HSA, AGE-HSA or CML-HSA were diluted 2:1 to a final concentration of 100 μg/ml with 2,4,6-Trinitrobenzene sulfonic acid (TNBSA) 0.01% in sodium carbonate buffer pH 8.5 (P2297; Sigma) and incubated 2 hours at 37°C. The reaction was terminated via dilution of the mixture 3:1:0.5 with SDS 10% and HCl 1 M, respectively. The absorbance at 420 nm was measured in a plate reader (SpectraMax Plus 384; Molecular Device), and the free amines were determined using a glycine standard curved under the same conditions. The percentage of modification was calculated based of the number of free primary amines present in HSA: 59 lysine and 24 arginine residues [21].

### Immunoblotting

For direct detection of protein levels, whole-cell lysates solubilized in loading buffer, which contained 10 mM Tris pH 6.8, 2% SDS (15525–017; Invitrogen), 0.01% Bromophenol Blue (B0126; Sigma), 10% Glycerol (G33-500; Fisher) and 0.1 M DTT (D9779; Sigma), were used for SDS-polyacrylamide gel electrophoresis. SDS-PAGE was conducted on 10% polyacrylamide (1500; Calbiochem) Tris-glycine gels and transferred to polyvinylidene difluoride membranes (NEF1002; PerkinElmer) for immunoblotting according to standard protocols. Immunoblots were performed using the following antibodies: CML (ab27684; Abcam), RAGE (MAB5328, Millipore), phosphorylated ERK (4370; Cell Signaling), ERK (4695; Cell Signaling), phosphorylated p38 (4631; Cell Signaling), p38 (9212; Cell Signaling), phosphorylated JNK/SAPK (4668; Cell Signaling), JNK/SAPK (9258; Cell Signaling), phosphorylated Ser473 AKT (9271; Cell Signaling), AKT (8272; Cell Signaling), Vasopressin Type 1A receptor (Ab3506P; Millipore), SM-α-actin (ab5694; Abcam), SM-MHC (ab53219; Abcam), MyoC (ab22073; Abcam), SM-22α (ab14106; Abcam), phosphorylated Ser19 MLC (3671, Cell Signaling) and Tubulin (T5293, Sigma). Primary antibodies were detected with horseradish peroxidase-coupled rat (ab6734; Abcam), mouse (A-9044; Sigma) or rabbit (ab6721; Abcam) secondary antibodies using Pierce ECL 2 Western Blotting Substrate (80196, Pierce). Chemiluminescence was detected with Amersham Hyperfilm ECL (28906839, GE Healthcare) and quantified (Gel Analyzer 2010a). The relative quantification of the protein levels was performed by dividing the intensity of the band that corresponded to the protein of interest by the intensity of the band specified as the loading control protein.

### NF-κB/GFP assay

Twenty-four hours prior to cell stimulation, 70% confluent A7r5 cells, grown in glass-bottom 24-well plates (P24-1.5H-N, In Vitro Scientific), were transfected with Cignal Reporter Assay plasmids (CCS-031G, Qiagen) using Lipofectamine 2000 (52887, Invitrogen) according to the manufacturer’s procedure. Briefly, Lipofectamine 2000 was mixed in a 1:9 ratio with Opti-MEM (31985–070; Gibco) and incubated for five min. Two hundred fifty ng of negative, positive or assay plasmids were mixed with the same volume of opti-MEM used for the Lipofectamine 2000. The DNA mixture was subsequently added to the diluted Lipofectamine 2000 solution and incubated for 20 min. The cells were washed with PBS, and the cell media was replaced with DMEM that contained only 10% FBS and NEAA for 10 times the volumes used to dilute the Lipofectamine 2000. After the incubation, the transfection mixture was added to the cell and mixed. Twenty-four hours after the transfection, the cells were washed with PBS, starved with DMEM that contained only NEAA and stimulated with PBS, 1 mg/ml AGE-HSA or 1 mg/ml CML-HSA for 24 hours. GFP fluorescence was determined via fluorescence microscopy using an inverted epifluorescence microscope (Axio Observer Z1, Carl Zeiss, Germany) equipped with an AxioCam MRm camera (Carl Zeiss, Germany). Pictures were taken with a 20x objective and an Endow GFP band pass filter (41017, Chroma Technology Inc.) with excitation at 470/40 nm and emission at 525/50 nm. Micrographs were acquired under a constant exposure time and presented under standardized luminosity and saturation attributes. Fluorescence quantification was achieved with ImageJ software [22]; see the *Microscopy fluorescence image analysis* section for additional details.

### Actin labelling and fluorescence microscopy

Cells grown on 25 mm glass coverslips were treated 24 hours with PBS, 1 mg/ml AGE-HSA or 1 mg/ml CML-HSA. The cells were treated with PBS or 200 nM AVP five min prior to fixation with 4.0% paraformaldehyde (G6403, Sigma) in PBS for 10 min and at room temperature. The fixed cells were washed with PBS for five min and permeabilized with 0.1% Triton X-100 (807426; MP Biomedicals) in PBS for one min. The cells were then incubated with the blocking solution Image-iT (I36933, Invitrogen) for 30 min prior to incubation with Phalloidin/Texas Red (T7471, Invitrogen) and Hoechst (B2261, Sigma-Aldrich) for 30 min. The coverslips were then mounted on microscope slides using Prolong Gold anti-fade reagent (P39634, Invitrogen). An inverted epifluorescence microscope (Axio Observer Z1, Carl Zeiss) equipped with an AxioCam MRm camera (Carl Zeiss) was used for fluorescence acquisition. Band pass filters with excitation at 560/40 nm and emission at 630/60 nm were used for Texas-Red (Chroma Technology Inc.). Band pass filters with excitation at 365/40 nm and emission at 445/50 nm were used for Hoechst (Carl Zeiss). Micrographs were acquired under a constant exposure time and presented under standardized luminosity and saturation attributes. Fluorescence quantification was achieved with ImageJ software [22]; see the *Microscopy fluorescence image analysis* section for additional details.

### Microscopy fluorescence image analysis

Micrographs acquired under a constant exposure time were analyzed under standardized luminosity and saturation attributes using ImageJ software. First, the background noise was removed using the Remove Background algorithm with a setting of 1000 pixels. Then, the white noise contained in the images was also evaluated via measurement of the grey value distribution of an area that did not contain fluorescence. The maximum grey values of 40 for the eGFP filter and 100 for the Texas Red filter were standardly used to remove white noise from all images. The density of fluorescence was subsequently measured via integration of the grey values of positive fluorescence pixels for each picture and then used for statistical and data analysis.

### Quantitative real-time polymerase chain reaction

Total mRNA extractions were performed on cell pellets using TRIzol (15596026; Invitrogen) with chloroform (9180–01; J.T. Baker), following the manufacturer’s protocol. The aqueous layer was recovered, mixed with one volume of 70% ethanol and directly applied to an RNeasy Mini Kit column (74104; Qiagen). DNAse treatment on the column and total RNA recovery were performed according to the the manufacturer’s protocol. The mRNA quality and presence of contaminating genomic DNA were verified as previously described [23]. The mRNA integrity was assessed with an Agilent 2100 Bioanalyzer (Agilent Technologies). Reverse transcription was performed on 2.2 μg of total RNA with Transcriptor reverse transcriptase, random hexamers, dNTPs (04897030001; Roche Diagnostics) and 10 units of RNAseOUT (10777–019; Invitrogen) following the manufacturer’s protocol in a total volume of 20 μl. All forward and reverse primers were individually resuspended to 20–100 μM stock solution in Tris-EDTA buffer (11-05-01-05; IDT) and diluted as a primer pair to 1 μM in RNase DNase-free water (11-05-01-04; IDT). Quantitative PCR (qPCR) reactions were performed in 10 μl in 96-well plates on a CFX-96 thermocycler (BioRad) with 5 μL of 2X iTaq Universal SYBR Green Supermix (172–5120; BioRad), 10 ng (3 μl) of cDNA, and 200 nM of final (2 μl) primer pair solutions. The following cycling conditions were used: 3 min at 95°C; 50 cycles: 15 sec at 95°C, 30 sec at 60°C, and 30 sec at 72°C. The relative expression levels were calculated using the qBASE framework [24] and the housekeeping genes Tubb5, Rpl19 and Pum1 for rat cDNA. Primer design and validation were evaluated as previously described [23]. In every qPCR run, a no-template control was performed for each primer pair, which were consistently negative. All primer sequences are available in S1 Table. All primer design and mRNA experiments were conducted by the Laboratoire de Génomique Fonctionnelle de l’Université de Sherbrooke.

### Atomic force microscopy (AFM) measurements and analysis

Live cell AFM measurements were performed on a custom-made force measurement device, based on the design and operation of an AFM [25]. Measurements were conducted at room temperature with an AFM mounted on an inverted microscope (Axio Observer Z1, Carl Zeiss) equipped with an AxioCam camera, which enabled the precise positioning of the cantilever tip on the cell. All force measurements were performed with standard silicon nitride cantilevers and a nominal spring constant of 0.02–0.77 N/m mounted with a cylinder tip (PL2-CONTR-SPL, NANOSENSORS). Prior to each experiment, the cantilever was calibrated using thermal noise amplitude analysis [26]. Indentation experiments were performed at a constant indentation depth of 1500 nm and a constant rate of 1.5 μm/s through all experiments. These parameters enabled the production of a sufficient number of force traces to follow the Young’s Modulus variations in time and properly probe the cell mechanical response; previous work by Rotsch and Radmacher demonstrated that the response is determined, to a large extent, by the underlying cytoskeleton, which can be probed by AFM using a range of a few micrometers [27]. After reaching confluence, the cells grown in 6 cm petri dishes were starved and stimulated for 24 hours with 1 mg/ml AGE-HSA or 1 mg/ml CML-HSA or PBS as a control. For the stiffness measurement, the cells were placed under the AFM, and the tip was positioned over the cell body using an x-y piezoelectric stage. The basal cell rigidity was calculated from three indentation curves recorded at the perinuclear region for approximately 15 cells per petri dish. For stimulated cell rigidity measurements, the experiment was designed to record three indentation curves at the perinuclear region of three cells per petri dish before and after stimulation with PBS or 200 nM Arginine-Vasopressin for five min.

To determine the cell rigidity, an experimental contact point between the tip and the cell surface was assigned at an indentation force three times higher than the noise present in the initial region of the force curve. Using multiple potential contact points, which started 50 nm before and after the experimental contact point with increments of 5 nm, the data were iteratively fitted into the Hertz model to calculate the Young’s modulus (*E*); the calibrated spring constant (*k*) and a Poisson ratio (*v*) of 0.5 were used until the root mean square deviation (RMSD) of the fit was three times greater than the experimental noise. For a cylindrical tip, the relation between the applied force *F* and Young’s modulus *E* is provided by *F = 2aE/((1-v^2))d*, where *a* is the radius of the cylinder, and *d* represents the indentation depth, which typically ranged between 200–300 nm. *E* was calculated from the fit that contained the most data points and the lowest RMSD variation.

The cell height was determined from three sets of indentation curves performed on both the petri dish and the apical part of multiple nearby cells. This procedure was repeated for three different regions in multiple petri dishes treated with PBS, 1 mg/ml AGE-HSA or 1 mg/ml CML-HSA. The contact points on these curves were determined using the curve noise as previously described. The cell heights were calculated from the difference between the cell and petri contact points.

### Calcium measurements

Fluorescence from Fura-2-loaded cells was monitored as previously described [28]. Briefly, A7r5 cells grown on poly-L-Lysine (P4707; Sigma) coated coverslips were starved and stimulated with PBS, 1 mg/ml AGE-HSA or 1 mg/ml CML-HSA. The cells were washed twice with HBSS (20 mM HEPES, 120 mM NaCl, 5.3 mM KCl_2_, 3 mM CaCl_2_, 800 nM MgSO_4_, and 5 mM Glucose) and loaded with 2μM Fura-2/AM for 20 min at room temperature in the dark. The cells were washed with HBSS and incubated for an additional 20 min at room temperature in the dark to ensure the probe de-esterification. Coverslips were placed in a circular open-bottom chamber and were mounted on the stage of an Olympus microscope (Roper Scientific) controlled by a METAFLUOR 6.1 digital imaging and photometry system (Universal Imaging). This system enables the simultaneous acquisition of images of custom regions on interest per field of view (10–20 cells). A typical experiment corresponds to 60 sec of stabilization in HBSS followed by a 60 sec stabilization period, in which the extracellular calcium was removed by changing the media with HBSS prepared without CaCl_2_ and with 0.5 mM EGTA. The cells were then stimulated with 200 nM AVP to measure the intracellular calcium release from the sarcoplasmic reticulum. When the signal returned to baseline (approximately 180 sec), the media were changed back to HBSS that contained Calcium+ for an additional 150 sec to measure the extracellular calcium entry.

Changes in the cytosolic calcium concentration were determined via ratiometric fluorescence measured at 510 nm from 340/380 nm excitations [29]. The ratio of the fluorescence intensity was calculated from individual images and calibrated in a saturating Calcium+ environment as previously described [28,30–32]. All experiments were performed at room temperature, and the results are expressed as the free intracellular calcium concentrations as a function of time.

### Statistical analysis

The data are shown as the mean ± standard error of the mean (SEM). All statistical analyses were conducted using Instat 2.0 (GraphPad). Briefly, the data were first tested for normality using the Shapiro–Wilk test, and the appropriate parametric or non-parametric tests were conducted. For multiple group comparisons, analysis of variance (ANOVA) followed by the proper *post hoc* test was applied. See figure legends for additional details. Statistical significance was assumed at *p* ≤ 0.05.

## Results

### Glycation of HSA *in vitro*

To assess the degree of *in vitro* glycation of HSA, we measured the extent of the modifications of amino acids that contained primary amines. Figure B in S1 File indicates that untreated recombinant HSA has 18.8±0.1% of primary amines modified, whereas HSA incubated with glucose (AGE-HSA) is more extensively modified to a level as high as 81.6±0.9%. With CML-HSA, we identified more than 99.8±2.8% of the modified primary amines. To confirm the AGE formation in our preparations, we demonstrated the presence of CML adducts using a specific antibody. Figure C in S1 File confirms that CML-HSA predominately contains the CML adduct, and it was also identified in lower amounts in the HSA-AGE preparation, as well as the untreated HSA. These results demonstrate that AGE-HSA and CML-HSA are extensively glycated proteins suitable for the investigation of RAGE activation *in cellulo*.

### A7r5 cells as a model of RAGE activation in VSMC

To establish that rat aortic VSMC (A7r5) are a suitable model for the investigation of the impact of RAGE signaling on the smooth muscle cell functional phenotype, we first confirmed the expression of RAGE in this cell line (Figure A in S2 File). RAGE can activate multiple signaling pathways, including the PI3K/AKT kinases and the protein members MAPK family ERK1/2, JNK and p38 [9]; to confirm RAGE functionality in this cell model, we measured the RAGE-dependent signaling of these pathways in A7r5 cells stimulated by AGE-HSA and CML-HSA. Figures A-H in S3 File indicates that AGE modulates MAPK ERK 1/2, p38 and JNK, as well as AKT, which suggests RAGE is functional in A7r5 cells through its coupling to downstream signaling pathways. Finally, G-protein-coupled receptors (GPCRs), such as the Vasopressin type 1 receptor (V1R), are coupled to Gαq/11 G-proteins and signals primarily through the downstream effectors phospholipase C and protein kinase C [33] to regulate cellular contraction and vascular tone. To use V1R activation as a functional contractile assay to determine the phenotype status of VSMC, its expression was also confirmed in the A7r5 model (Figure B in S2 File). Therefore, we confirmed that the A7r5 cell line is a suitable model to investigate the effect of RAGE activation on the contractile phenotype and function of VSMC.

### RAGE-dependent NF-κB activity in VSMC

**Fig 1:**
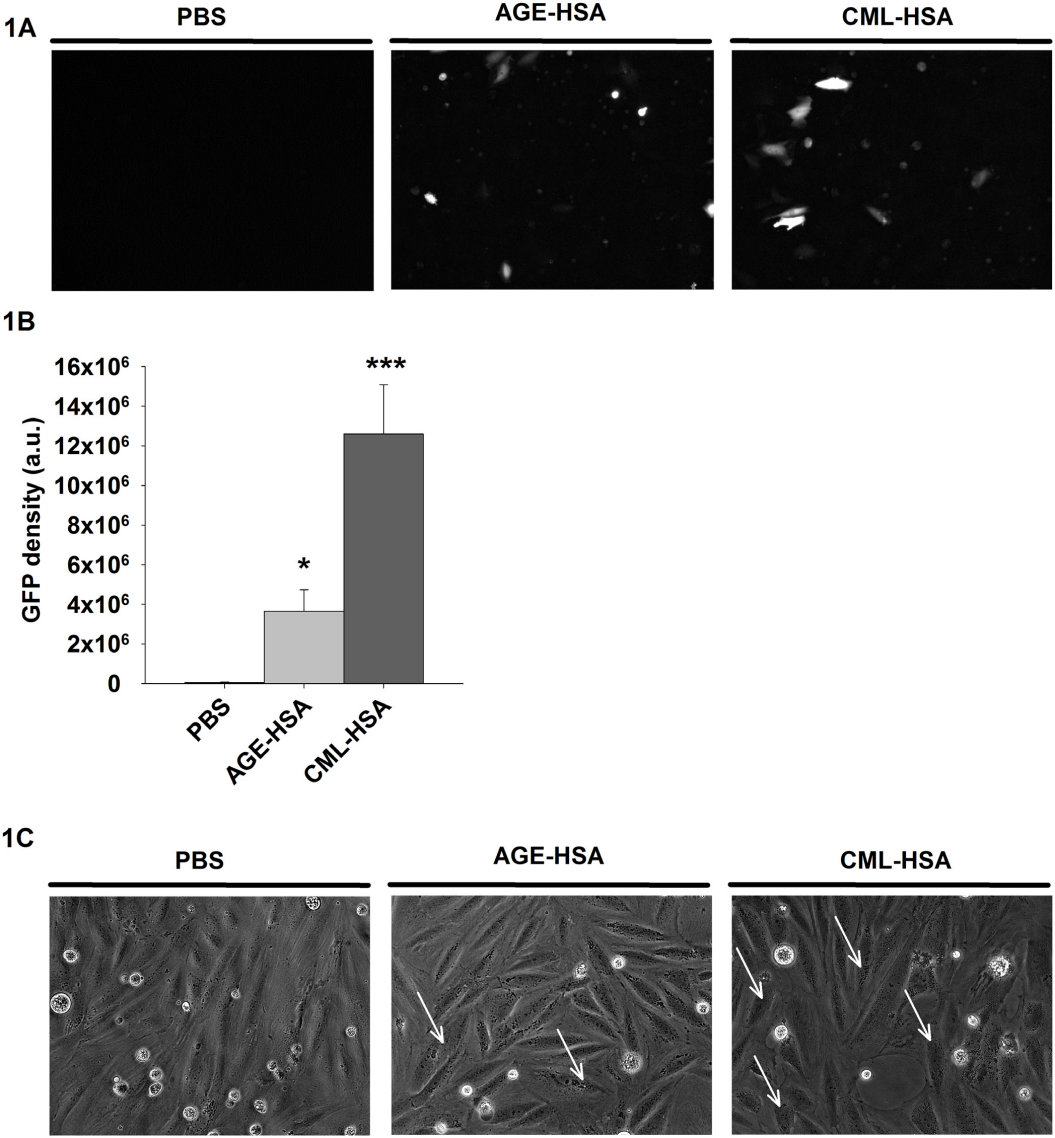
RAGE stimulation increases NF-κB activity. A, Representative epifluorescence micrographs (20X) and (B) Histogram of the average GFP fluorescence intensities indicate NF-κB dependent GFP expression in A7r5 cells stimulated for 24 hours with PBS, 1 mg/ml AGE-HSA or 1 mg/ml CML-HSA. Each bar represents 10–12 independent experiments. * *p* < 0.05 compared with PBS using one-way ANOVA followed by Dunn’s post-test. C, Representative phase contrast micrographs (20X) indicate the morphological effects of stimulations with PBS, 1 mg/ml AGE-HSA or 1 mg/ml CML-HSA in A7r5 cells for 24 hours (n = 6).

As presented in the introduction, several lines of evidence have demonstrated that NF-κB signaling in VSMC is detrimental to the expression of their contractile phenotype. However, little is known regarding the NF-kB activity dependence of RAGE signaling on the contractile phenotype and function of VSMC. Thus, to determine if VSMC are sensitive to NF-κB-mediated RAGE signaling, we first quantified NF-κB activity via the measurement of the GFP signal in A7r5 cells stimulated with AGE-HSA, CML-HSA or PBS and transfected with a GFP-based gene reporter plasmid for NF-κB. Fig 1A and 1B indicate that A7r5 cells treated with AGE-HSA or CML-HSA exhibit increased NF-κB activities (3.6±1,1x10^6^ and 12.6±2,5x10^6^ a.u., respectively) compared to PBS treated cells (0.5±0.4x10^6^ a.u.) as demonstrated by the increased GFP expression. These results validate that RAGE activation in VSMC is associated with increased NF-κB activity. We subsequently determined whether AGE-HSA, CML-HSA or PBS stimulations resulted in morphological changes in the A7r5 cell line. Fig 1C indicates that A7r5 cells stimulated with either AGE-HSA (central panel) or CML-HSA (right panel) do not significantly differ in morphology compared with the cells treated with PBS (left panel). However, increased granularity, which could be associated with cellular stress and P-body formation [34], has been observed in A7r5 cells treated with AGE-containing HSA (white arrows, central and right panels); these findings suggest that VSMC are sensitive to RAGE signaling.

### Modification of the transcriptome and protein expression by RAGE signaling

**Fig 2:**
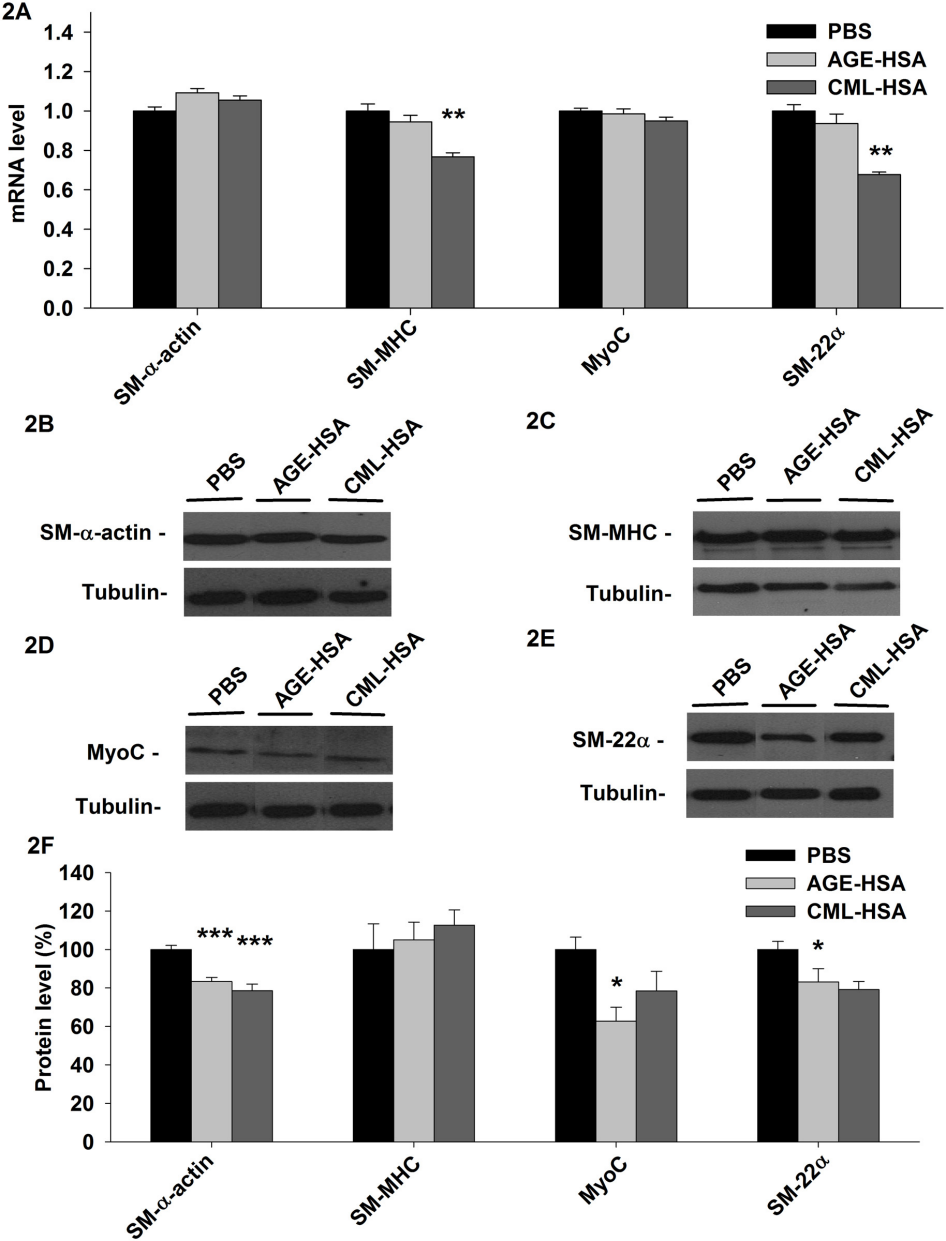
RAGE signaling affects the RNA and protein levels of different key proteins involved in the VSMC contractile phenotype. A, Histogram of the mRNA levels of SM-α-actin, SM-MHC, MyoC and SM-22α measured via qPCR, immunoblots indicate (B) SM-α-actin, (C) SM-MHC, (D) MyoC and (E) SM-22α protein levels and (F) Semi-quantitative analysis of the indicated protein levels in A7r5 cells stimulated 24 hours with 1 mg/ml AGE-HSA or 1 mg/ml CML-HSA. Each bar represents 10–12 independent experiments. For mRNA, the data are expressed relative to PBS. For the protein levels, the data were normalized based on the corresponding tubulin loading control and then expressed as a % relative to PBS. * *p* < 0.05 compared with PBS using one-way ANOVA followed by Dunett’s post-test.

To examine the potential impact of RAGE signaling on the contractile phenotype of VSMC, we measured the changes in the mRNA level of key genes involved in the cell contraction machinery (SM-α-actin and SM-MHC) or responsible for the maintenance and regulation of the cell contractile phenotype (MyoC and SM-22α) in A7r5 cells stimulated with AGE-HSA, CML-HSA or PBS. Fig 2A indicates that significant changes in mRNA are present only in cells treated with CML-HSA. More precisely, the SM-MHC mRNA level was decreased (0.884±0.023) compared with the cells treated with PBS (1.152±0.041). Similarly, the SM-22α mRNA levels were also lower (0.795±0.016) in the cells treated with CML-HSA compared to the control (1.174±0.037).

To identify the impact of the changes observed in the transcriptome of VSMC stimulated with AGE, we also measured the changes in the protein levels of SM-α-actin, SM-MHC, MyoC and SM-22α. Fig 2B and 2F indicate that both AGE-HSA (77.6±2.0%) and CML-HSA (73.3±3.1%) treatments led to a reduction in SM-α-actin levels compared with PBS (93.2±2.0%). In contrast, the SM-MHC levels (Fig 2C and 2F) were not affected by either RAGE ligand. Only AGE-HSA (50.4±5.8%) significantly reduced the MyoC levels (Fig 2D and 2F), although a similar tendency could be observed with CML-HSA (63.0±8.3%) compared with PBS (80.3±5.2%); only the CML-HSA treatment induced a significant reduction in the SM-22α protein levels (66.6±3.6%) compared with PBS (84.2±3.6%). Finally, AGE-HSA (70.0±5.8%) appeared to have a similar effect (Fig 2E and 2F). These results suggest that RAGE signaling in VSMC modulates the transcriptome and protein expression typically associated with its contractile phenotype.

### RAGE-dependent modulation of VSMC mechanical properties

**Fig 3:**
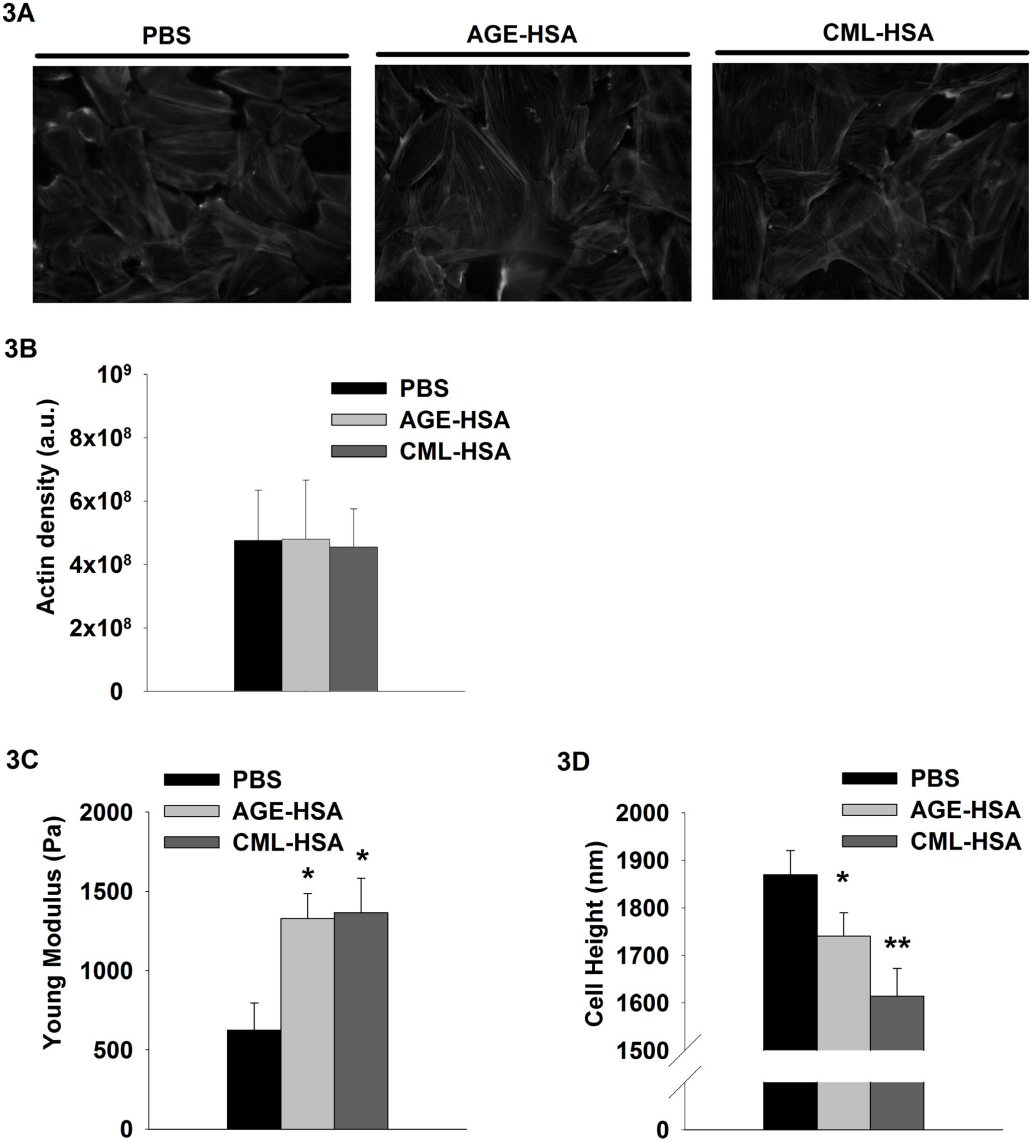
Impact of RAGE signaling on cell mechanical properties. A, Representative epifluorescence micrographs (40X) of Phalloidine/TexasRED stained cells that exhibit actin organization, (B) histogram of the average TexasRED fluorescence that corresponds to the actin density, (C) histogram indicates cell rigidity (Young Modulus) and (D) cell heights measured by AFM of A7r5 cells stimulated for 24 hours with PBS, 1 mg/ml AGE-HSA or 1 mg/ml CML-HSA. Each bar represents 12–15 independent experiments. * *p* < 0.05 compared with PBS using one-way ANOVA followed by Dunn’s post-test.

Mechanical characteristics, such as cytoskeletal organization, cell rigidity and the ability of the cell to contract, are relevant to the investigation of VSMC functionality, which, in turn, depends on the expression of its contractile phenotype. To investigate the potential impact of RAGE signaling on the contractile function of VSMC, we first determined the effects of AGE-HSA and CML-HSA stimulations of A7r5 cells on their actin structure and the overall stiffness of the cell body. Previous reports from our laboratory and other groups have demonstrated that the contractile machinery activity can be measured through time-resolved changes in cell rigidity [4,25]. The findings indicate that AGE-HSA and CML-HSA did not affect the general structure (Fig 3A) or the density of the actin cytoskeleton (Fig 3B). However, Fig 3C indicates a dramatic increase in the basal cell rigidity from 623±171 Pa for PBS to 1328±157 Pa for AGE-HSA and 1366±215 Pa for CML-HSA. Therefore, the cells stimulated with AGE-HSA or CML-HSA offer more resistance to deformation compared with their PBS counterpart, which suggests increased intracellular tension associated with cell contraction. Interestingly, we measured a reduced cell height with respect to the substrate (Fig 3D), which could be consistent with a potential increase in intracellular tension. Compared with the cells treated with PBS that exhibited a nominal height of 1869±51 nm, the cells treated with either AGE-HSA or CML-has exhibited a significantly reduced height (1614±58 and 1740±49 nm, respectively).

**Fig 4:**
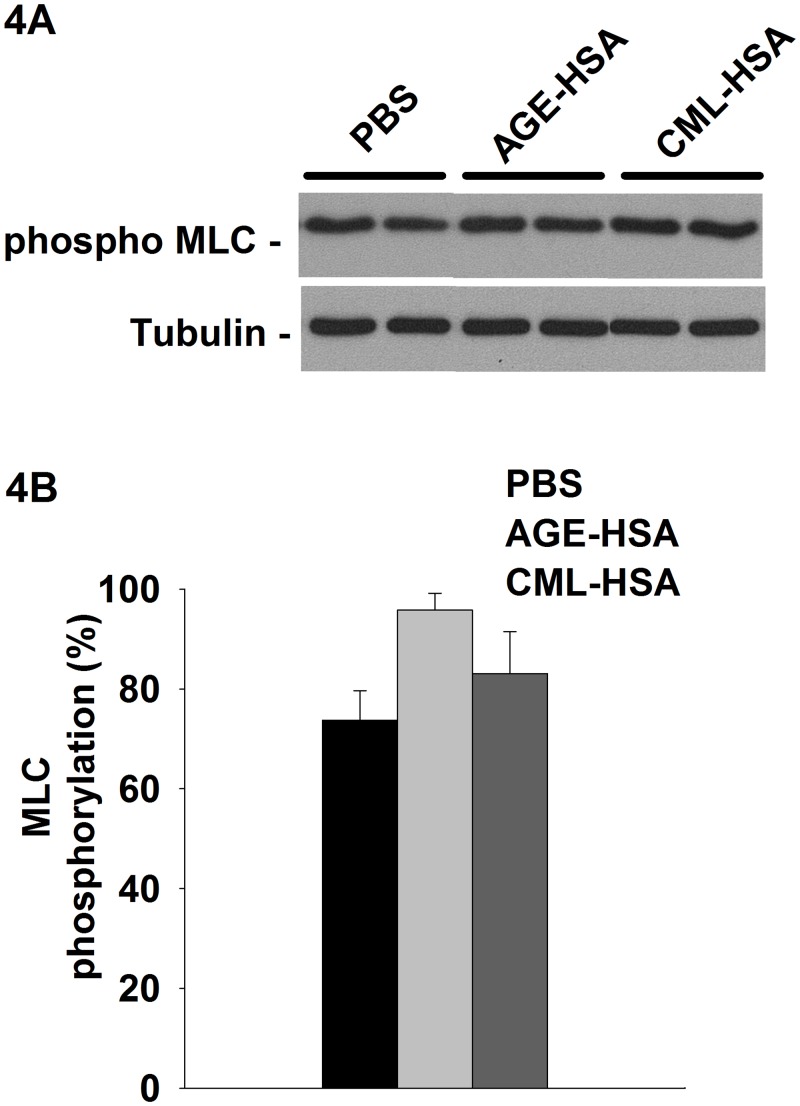
RAGE signaling does not significantly affect basal myosin phosphorylation. A, Immunoblots indicate the phosphorylated levels of MLC and (B) semi-quantitative analysis of the pMLC activity in A7r5 cells stimulated 24 hours with 1 mg/ml AGE-HSA or 1 mg/ml CML-HSA. Each bar represents 9–12 independent experiments. The data were normalized based on the corresponding tubulin loading control and then expressed as % relative to the maximum phosphorylation value.

VSMC contraction is mainly governed by actin-myosin-mediated motor activity which is regulated by the phosphorylation of its light chain (MLC) [5] catalyzed by Myosin Light Chain Kinase (MLCK) or Rho-activated Kinase. Therefore, as part of the investigation of the potential impact of RAGE stimulation on the contractile function of VSMC, we assessed the impact of AGE-HSA and CML-HSA stimulation on the basal activity of myosin by measuring the phosphorylated MLC levels. Overall, even if a tendency for an increased basal activity of myosin could be observed for both AGE, these experiments (Fig 4A and 4B) indicated there were no significant differences among PBS (73.7±5.9%), AGE-HSA (95.8±3.4%) and CML-HSA (83.1±8.4%). Thus, these results indicate that RAGE signaling modulates VSMC mechanical properties.

### Regulation of calcium homeostasis and signaling by RAGE activation

**Fig 5:**
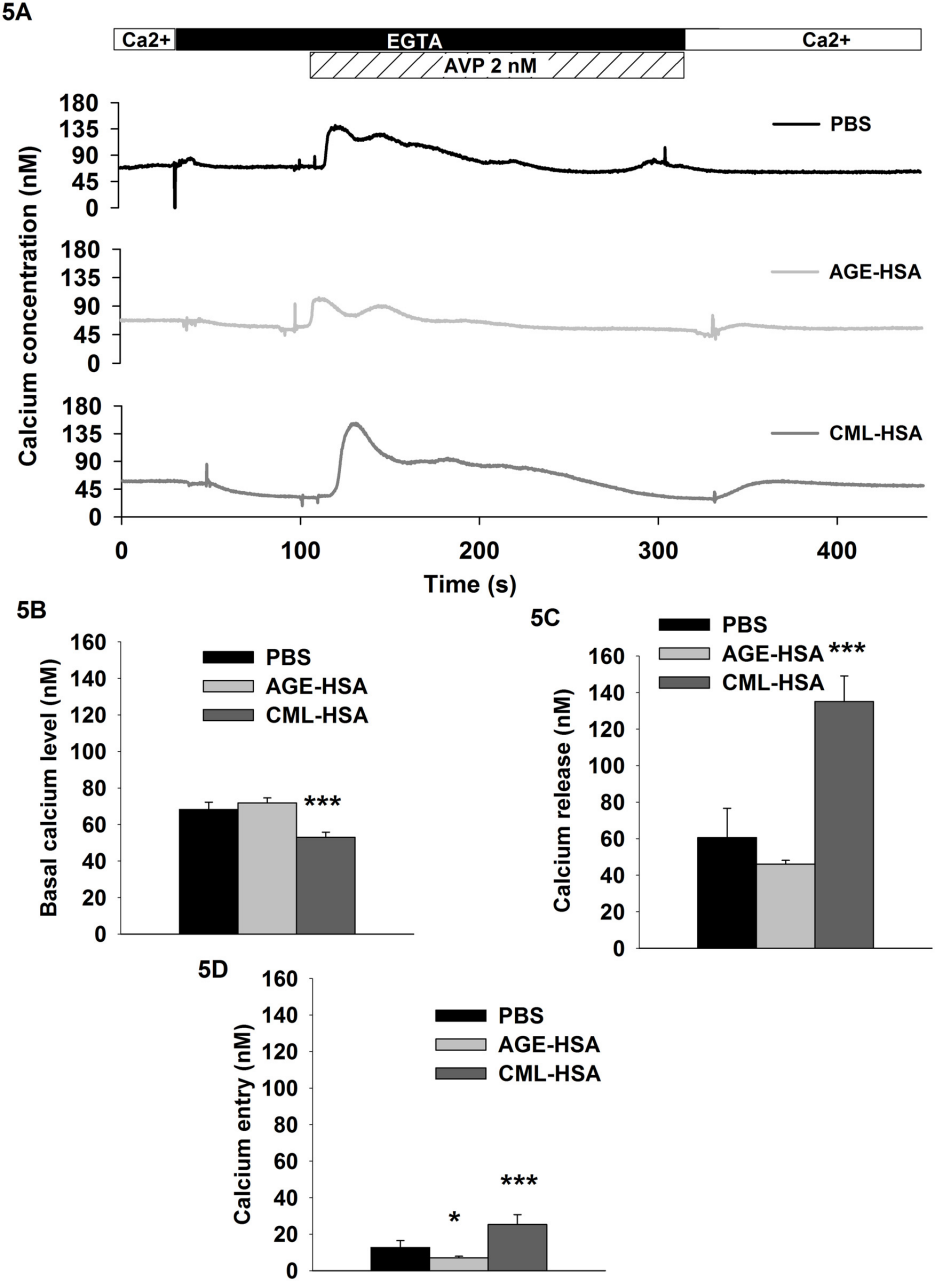
RAGE signaling increases V1R/Gq-dependent calcium signaling. A, Typical calcium measurement kinetics obtained with FURA-2 and histograms indicate (B) basal calcium levels, (C) Gq-dependent calcium release and (D) calcium entry in A7r5 cells stimulated for 24 hours with PBS, 1 mg/ml AGE-HSA or 1 mg/ml CML-HSA and challenged with 2 nM AVP. Each bar represents 9–12 independent experiments. * *p* < 0.05 compared with PBS using one-way ANOVA followed by Dunnett’s post-test.

To determine the effect of RAGE signaling on the signaling capacity of VSMC associated with cell contraction, we measured the V1R-dependent calcium response in A7r5 cells pretreated with AGE-HSA, CML-HSA or PBS and stimulated with AVP. The activation of V1aR leads to Gαq/11-dependent intracellular calcium mobilization, which is responsible for the activation of the CAMK/MLCK pathway and the concomitant contraction in VSMC [35,36]. Fig 5A and 5B indicate that in the absence of extracellular calcium, CML-HSA pretreatment affected the basal calcium levels (52.9±2.8 nM) compared with PBS (68.2±3.9 nM). Moreover, Fig 5A and 5C indicate that AVP-induced calcium released from the sarcoplasmic reticulum was also increased by CML-HSA pretreatment (135±14 nM) compared with PBS (60.6±15.9 nM). Remarkably, whereas calcium entry (Fig 5A and 5D) was decreased in the cells pretreated with AGE-HSA (7.0±0.8 nM), it was increased in the cells pretreated with CML-HSA (25.2±5.4 nM) compared with the control (12.6±3.8 nM). These results demonstrate that sustained RAGE signaling in VSMC affects the cellular calcium homeostasis and agonist-induced calcium response.

### Alteration of VSMC contractile activity by AGE-dependent RAGE signaling

**Fig 6:**
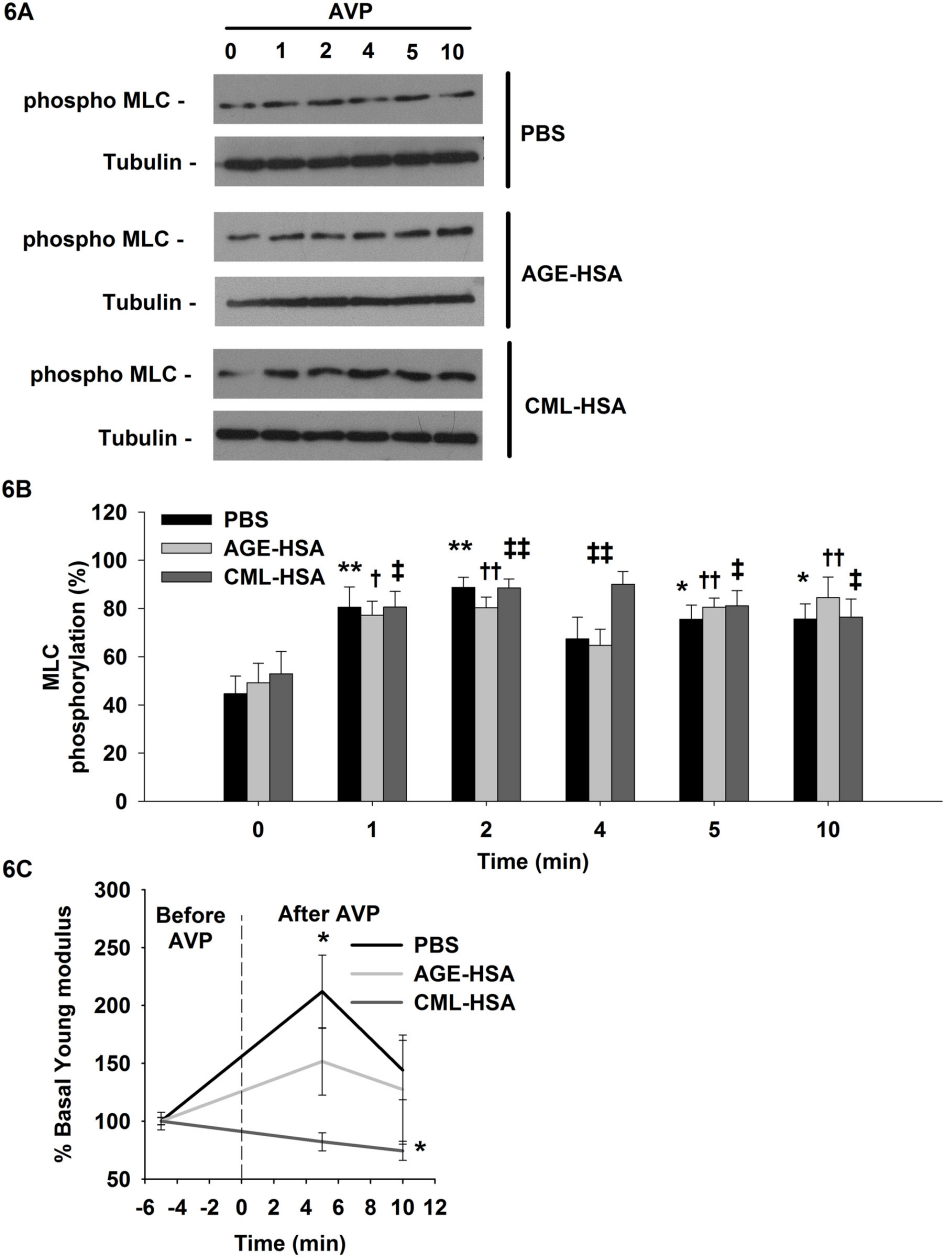
RAGE signaling affects myosin phosphorylation and cell contraction induced by AVP stimulation. **A**, immunoblots indicate the phosphorylated levels of MLC and (**B**) semi-quantitative analysis of the pMLC activity in A7r5 cells stimulated 24 hours with 1 mg/ml AGE-HSA or 1 mg/ml CML-HSA. Each bar represents 11–12 independent experiments, and tubulin was used as the loading control for MLC activity determination. The data were normalized based on the corresponding tubulin loading control and then expressed as a % relative to the maximum phosphorylation value. * *p* < 0.05 compared with the respective control (0 min) using one-way ANOVA followed by Bonferroni post-test. **C**, Graph indicates the changes in Young modulus as measured by AFM. A7r5 cells stimulated 24 hours with 1 mg/ml AGE-HSA, 1 mg/ml CML-HSA or PBS were probed 5 min prior to stimulation to obtain basal values; the same cells were subsequently probed 5 min and 10 min after 200 nM AVP stimulation. The data are expressed as the % changes in the cell rigidity compared with the respective control (-5 min). Each condition represents 12–15 independent experiments. * *p* < 0.05 compared with the control (-5 min) using one-way ANOVA followed by Dunn’s post-test.

As previously discussed, MLC phosphorylation can be associated with cell contractile activity. To determine the impact of RAGE activation on this biomarker of cell contraction in VSMC, we monitored the changes in MLC phosphorylation levels in A7r5 cells pretreated with AGE-HSA, CML-HSA or PBS and stimulated with AVP. As expected, the cellular MLC activity increased in all conditions after AVP stimulation (Fig 6A and 6B). The overall MLC activity was similar in the different conditions; however, it was slightly increased in the cells treated with CML-HSA. The phosphorylated MLC levels (90.0±5.2%) were significantly increased after 4 min of AVP stimulation compared with the AGE-HSA (64.7±6.7%) or PBS (67.3±9.0) pretreated cells.

The cell contractile activity can be positively correlated with rearrangements in the cell morphology and cytoskeletal structure or variations in cell rigidity; thus, we examined both the evolution of the actin organization and the Young Modulus of A7r5 cells preconditioned with AGE-HSA, CML-HSA or PBS before and after AVP stimulation. No contraction of the cell body was visually identified following AVP stimulation or for any condition, as demonstrated by the lack of rearrangement of the actin cytoskeleton (Figures A and B in S4 File.); only the control cells (211±31%) increased their rigidity (Fig 6C) after AVP stimulation compared with the cells pretreated with AGE-HSA (151±29%) or CML-HSA (82.1±7.8%). This important result suggests that RAGE activation interferes with the mechanical competency of VSMC.

## Discussion

Phenotypic modulation of VSMC plays a central role in vascular development and remodeling during disease. In response to vascular injury, VSMC de-differentiate to a proliferative/synthetic phenotype, which is involved in the various pathological states, such as atherosclerosis, neointimal hyperplasia and hypertension [37,38]. To our knowledge, limited attention has focused on the functional effect of RAGE activation in VSMC other than the work of Suga *et al*. [39] and Tanikawa et al. [40]; these findings indicate that RAGE activation in VSMC induces the expression of cell properties that could be assimilated to osteoblasts, which thereby establishes a link between RAGE activation and vascular calcification in diabetes. The present study is the first study to investigate the phenotypic and functional effects of endogenous RAGE activation on the contractile activity of VSMC. This study contributes novel data regarding the mechanical properties of cells that were or were not subjected to RAGE activation. We tested the effects of two ligands, AGE-HSA and CML-HSA, on many levels of the VSMC cellular responses to propose a stronger model to investigate RAGE activation.

In the investigation of RAGE signaling, it is common to obtain ligands from the incubation of albumin with glucose at temperatures greater than 37°C for durations of up to eight weeks [41]. A variety of AGE structures with different affinities for the RAGE receptor can be obtained from these preparations, which produce unique mixtures of AGE [41]. Variations in AGE preparation and perhaps the degree of CML modifications may explain the discrepancies identified in the doses and effects reported in previous studies. CML protein adducts are a major immunological epitope in proteins, such as albumin, subjected to the Maillard reaction [42], and the accumulation of CML-modified proteins *in vivo* has been reported in several human and animal tissues in normal and pathological conditions [43]. Here, we suggest that CML-HSA may represent a better ligand to investigate RAGE activation; consequently, we evaluated the effects of both AGE-HSA and CML-HSA on cell signaling, transcription factor activation, mRNA and protein expression, and functional cellular responses, such as cell contraction. As expected, our results indicated more consistency and stronger effects with CML-HSA stimulations, which suggest that CML-HSA represent a more suitable choice to investigate RAGE activation. Moreover, the various potencies in the identified effects are likely caused by the difference in the concentrations of CML adducts identified between the mixed AGE-HSA and pure CML-HSA preparations. In a limited number of experiments, AGE-HSA has exhibited opposing results to CML-HSA, which may be explained by considering that different levels of NF-κB activation can affect the cellular activity in different and potentially opposing ways. Increased NF-κB activity may promote apoptosis, whereas lower levels participate in the transcription of genes that favor cell survival [44]. Thus, we believe that the ability of both ligands to activate ERK 1/2 and AKT and induce NF-κB activation to largely different degrees could explain the discrepancies identified regarding VSMC differentiation and functionality.

VSMC differentiation is dependent on the expression of appropriate levels of smooth muscle contractile genes, whereas proliferating VSMC exhibit reduced expression levels of these genes. Proteins, such as SM-α-actin, SM-MHC and SM-22α, are established markers of functional smooth muscle cells with the contractile phenotype, which is finely regulated by a wide range of physiological cues [38]. The premise of our study is that RAGE activation contributes to vascular diseases by altering the VSMC contractile phenotype. A7r5 cells are derived from fetal rat thoracic and abdominal aortas and have been extensively used as a model to investigate the VSMC phenotype [45–47]. These cells are capable of generating action potentials that can travel from cell to cell through low resistance junctions and synthetize muscle type creatine phosphokinase, which are both markers of a differentiated and functional SMC [48]. In our study, we confirmed the validity of this VSMC model via the demonstration that A7r5 cells express extensive levels of SM-α-actin and SM-MHC mRNAs and proteins. We also demonstrated that A7r5 cells transcript and translate important levels of MyoC, which is a protein involved in the control of smooth muscle cells with the contractile phenotype [49], and SM-22α, which is a marker of smooth muscle cells with the contractile phenotype and a regulator of cell contraction [50]. However, more importantly, using a single cell-based mechanical assay, we demonstrated that A7r5 cells are mechanically competent and capable of contraction as observed with the increased rigidity induced by AVP.

RAGE activation leads to increased MAPK, PI3K/AKT and JAK/STAT activities, which are involved in NF-κB transcription factor activation. MAPK ERK 1/2 signaling in VSMC has been extensively studied and has been associated with increased cell proliferation, DNA synthesis and agonist-induced cell migration *in vitro* and *in vivo* [51]. MAPKs, JNK and p38 are stressed activated kinases involved in the adaptation to stress, such as inflammation, cell differentiation and apoptosis [52], whereas the PI3K/AKT pathway plays a key role in multiple cellular processes, such as glucose metabolism, apoptosis, cell proliferation, transcription and cell migration [53]. Here, we confirmed that A7r5 cells represent a valid VSMC model to investigate RAGE activation via the demonstration that A7r5 cells express endogenous levels of RAGE, and when exposed to AGE, their ERK 1/2, JNK p38, PI3K/AKT and NF- κB activities are modulated.

Previous reports that involve other receptors have demonstrated that NF-kB signaling interfered with the expression of the VSMC contractile phenotype [17]. Our results indicate that RAGE activation increased NF-κB activation in VSMC, which correlated with decreased biochemical markers of the VSMC contractile phenotype. The SM-α-actin, MyoC and SM-22α levels were decreased in the AGE-HSA and CML-HSA stimulated cells. We believe that this decrease is the result of MyoC degradation, as reported by other groups [54], because the mRNA levels were not affected by RAGE stimulation. However, the link between the lack of changes in the mRNA levels and the reduced protein expression of SM-α-actin is unclear and must be further investigated. Nevertheless, the increase in MyoC degradation clearly affected the SM-22α gene and protein expression in cells stimulated with both AGE. These results support our hypothesis that RAGE activation in VSMC interferes with their contractile phenotype by altering the expression of proteins involved in cell contraction regulation.

Interestingly, RAGE activation had no effect on the actin organization or density and no significant effect on the myosin activity in VSMC. However, the mechanical properties of A7r5 cells, such as cell rigidity and height, were affected by AGE-dependent RAGE signaling. Cell rigidity is an important phenotypic characteristic of a mechanically competent cell because it influences the cell’s ability to contract, migrate and proliferate [55]. It has been reported that undifferentiated cells [56] and invasive cancer cells [57] are often softer, which appears to facilitate their migration process. However, increased cell rigidity has been reported in aging cells [58] and many progressive diseases, such as hypertension, renal failure, cataracts, Alzheimer’s disease and complications associated with diabetes [59,60]. Here, we demonstrate for the first time that RAGE activation increases cell rigidity in a model of VSMC, which correlates with a lower cell height and the tendency in the increased basal phosphorylation of MLC; these findings suggest increased cellular tension and adhesion. Previous work has demonstrated that VSMC respond to mechanical forces in their environment, such as stretch applied to their extracellular matrix, by adapting the expression of genes and phenotypes [61]. Therefore, it is proposed that increased rigidity induced by RAGE activation in VSMC is responsible *in vivo* for changes in the phenotype of VSMC via alterations in the cell’s ability to respond to stretch forces. Consistent with the results presented in our study, an increase in the stiffness of the extracellular matrix of blood vessels has been previously associated with reduced vessel compliance to stretch forces that originate from blood flow and the promotion of a hypertensive state [62]. Therefore, increased stiffness of VSMC induced by RAGE activation could contribute in a similar way to the development of hypertension and vascular diseases in diabetics.

RAGE activation in VSMC, via alterations in the cell phenotype, ultimately interferes with their primary contractile function. Our results demonstrate that the biomarker of cell contraction (MLC phosphorylation), had its level and kinetic altered and calcium (a second messenger known to be involved in inducing cell contraction) was also released in greater amounts. Interestingly, it is well established that calcium signaling acts a regulator of gene expression in cells. It has recently been demonstrated that altered calcium entry in VSMC can promote the expression of an early-response c-fos gene and activated ERK 1/2. These events potentially lead to VSMC growth and proliferation [63]. Therefore, it is plausible that increased calcium entry, which is induced by RAGE activation in VSMC, leads to phenotypic alterations of VSMC *in vivo*, as well as a concomitant modulation of their ability to respond to contractile hormones. In this context, our results strongly suggest that RAGE activation participates in the hypertensive state in diabetes by also amplifying VSMC responses to vasopressive hormones, such as AVP.

A7r5 cell contraction has been previously reported indirectly using ultrasensitive microscopy and sophisticated image analysis methods, which indicated minimal reorganization of the contractile machinery following contraction [64]; these findings were confirmed by our results that indicated no detectable changes to actin structures or density during RAGE activation or concomitant AVP stimulations. We have previously reported that strong correlations exist between agonist-induced cell contraction and an increase in cell rigidity, as measured by AFM [25]. In our study, it should be noted that the time-course measurement of the Young modulus following VSMC stimulation with AVP can be assimilated to a direct monitoring of cell contraction. Following AVP stimulation, MLC is phosphorylated, which leads to an increase in the intracellular tension that is reflected in the increased Young’s modulus.

Here, we demonstrate that despite increased calcium signaling and MLC phosphorylation in VSMC exposed to AGE ligands and stimulated with AVP, the VSMC contractile ability is impaired. The cell rigidity of VSMC measured before and after the AVP stimulation did not increase in the A7r5 cells pretreated with AGE ligands. We have previously demonstrated that a correlation does not always exist between the MLC phosphorylation levels and cell contractile response; this discrepancy would typically indicate disturbances in the tightly regulated pathways involved in cell contraction [65]. Thus, the disturbances provoked by the decreased expression of contractile phenotype-associated proteins and regulators, such as SM-α-actin, MyoC and SM-22α, in VSMC could lead to an uncoupling between the signaling typically associated with cell contraction (i.e., increased calcium and MLC phosphorylation) and the mechanical contraction *per se*.

Substrate rigidity affects the actin polymerization status and the cell rigidity; hard substrates, such as the petri dishes used in our study, are well-known to promote actin structures and increase cell rigidity [66], which could mask the full impact of RAGE signaling on VSMC phenotype. Therefore, in VSMC grown on softer substrates, such as the one found in blood vessels, RAGE signaling could possibly have a larger effect on actin organization, basal rigidity and the contractile function of these cells.

Our results support the hypothesis that RAGE activation in VSMC interferes with the functions associated with their contractile phenotype. Interestingly, it has been previously demonstrated that RAGE activation by AGE in primary human aortic vascular smooth muscle cells induced cell calcification [40]. Similar to our results, VSMC treated with AGE for 12–72 hours exhibited significant increases in the markers associated with osteoblast functions, such as alkaline phosphatase or osteocalcin expression. In our study, we identified a loss of the contractile phenotype and function, which could be part of a continuum of events that ultimately lead to the expression of an osteoblast-like phenotype in these cells and promote vascular calcification in diabetes.

## Conclusion

**Fig 7:**
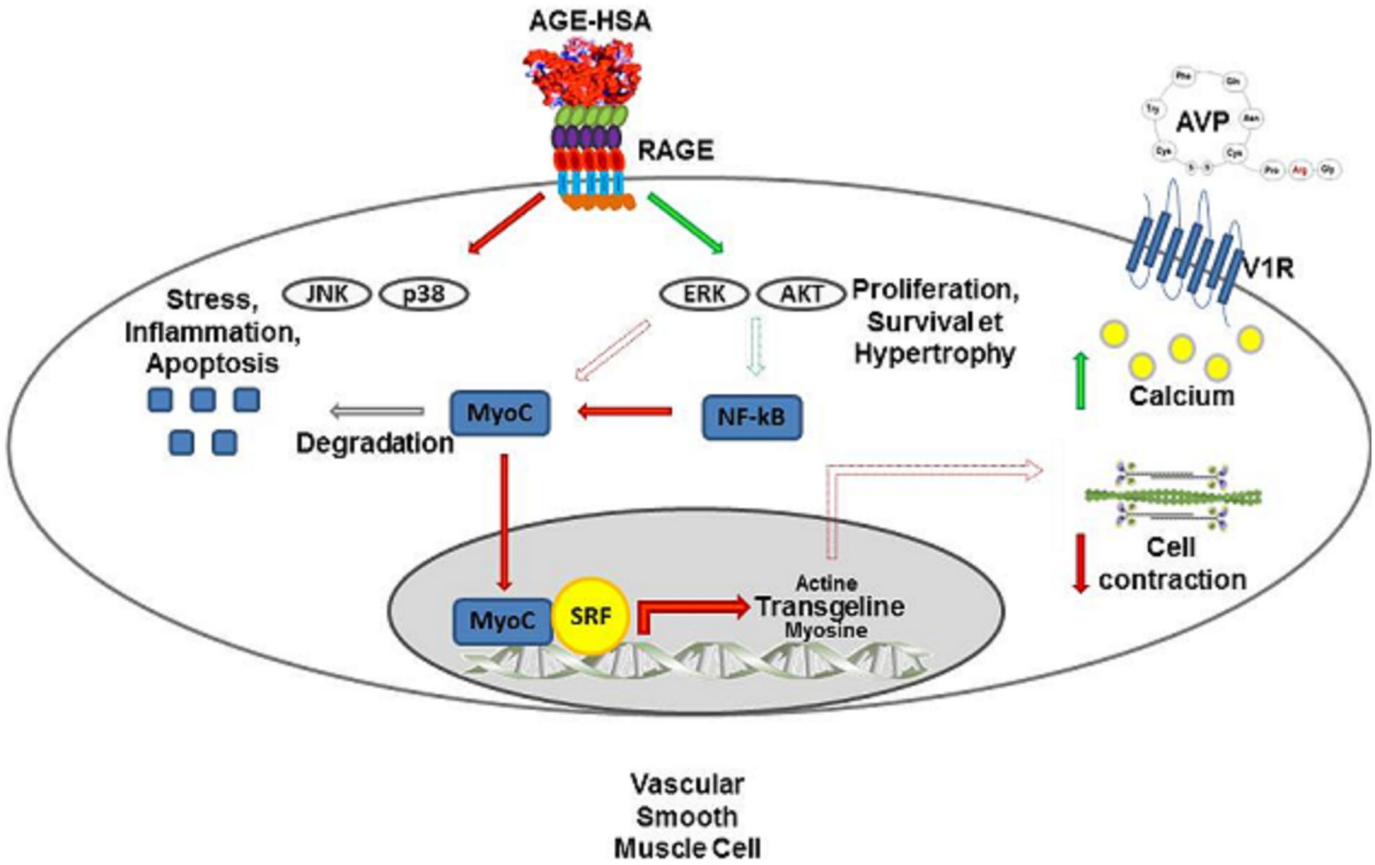
RAGE activation in VSMC interferes with their contractile phenotype and function. RAGE is expressed in VSMC A7r5 and fully functional. Its activation, using short term stimulation with AGE-HSA or CML-HSA, leads to increased ERK 1/2 and AKT signaling, whereas p38 and JNK/SAPK activities are decreased. Long term exposure to AGE increases NF-κB activity, which affects the protein levels of the contraction-related transcription factor MyoC and the mRNA and protein levels of SM-22α, a regulator of VSMC contraction. Finally, RAGE activation increases the overall cell rigidity, which is likely caused by changes in myosin activity. Interestingly, although RAGE stimulation amplifies calcium signaling and myosin activity in VSMC challenged with AVP, these cells lose their contractile capacity when measured via AFM.

RAGE activation participates in numerous pathophysiological conditions, such as Alzheimer’s disease, arthritis, many pulmonary diseases (acute lung injury, acute respiratory syndrome and asthma), sepsis and atherosclerosis [67]. Diabetes is an important risk factor for the development of cardiovascular diseases, such as atherosclerosis, coronary disease and cardiomyopathy [68], and individuals with diabetics have increased AGE levels [69,70]; thus, it has been proposed that a link may exist between RAGE and the increased vascular diseases in diabetics [9,71–73]. It has been demonstrated in diabetes that AGE contribute to impaired endothelial function and change vessel wall properties via the promotion of basement membrane thickening, increased vascular permeability, and the pro-thrombotic state, which result in damage to the retina, nephron, peripheral and central nervous systems and atherosclerosis [68]. Here, we provide further evidence that is summarized and illustrated in Fig 7; consistent with previous findings, we conclude that RAGE activation in VSMC is also likely a keystone component in the development of vascular diseases associated with diabetes via interference with the contractile phenotype of VSMC through the modification of their mechanical and functional properties.

## Supporting Information

S1 File *In vitro* glycation of HSA increases CML adducts. Human recombinant albumin was treated in order to produce AGE-HSA or CML-HSA. Figure A, A7r5 cell culture media containing AGE as revealed by immunobloting for CML adduct. These results confirm that glycation occurred *in vitro* and reveal that untreated HSA carries a certain degree of modification forbidding its use as an experimental control. Thus, PBS was used as a negative control in our study. Figure B, Degree of modification of amino acids containing primary amines using the TNBSA assay. The results show that untreated recombinant HSA has a degree of modification of 18.8±0.1% whereas HSA incubated with glucose (AGE-HSA) is extensively modified to 81.6±0.9%. Similarly, CML-HSA has more than 99.8±2.8% of its primary amines modified. Figure C, Presence of CML adducts in the different preparations by immunoblotting using a CML specific antibody. Results show that CML-HSA contains the CML adduct, which appears less prevalent in the AGE-HSA preparation and in the untreated HSA.
(TIF)

S2 File Expression levels of RAGE and V1R in A7r5 cells. Immunoblots showing the expression of (Figure A) RAGE and (Figure B) V1R in A7r5. Tubulin was used as loading control.
(TIF)

S3 File RAGE signaling activates MAPK and AKT signaling pathways. Immunoblots showing phosphorylated and total (Figure A) ERK 1/2, (Figure B) p38, (Figure C) JNK/SAPK or (Figure D) AKT proteins levels in A7r5 cells stimulated with (left panels) 1mg/ml AGE-HSA or (right panels) 1mg/ml CML-HSA at indicated times. Semi-quantitative analysis of (Figure E) ERK, (Figure F) p38, (Figure G) JNK/SAPK or (Figure H) AKT activation in A7r5 cells stimulated for indicated times with 1mg/ml AGE-HSA or 1 mg/ml CML-HSA (n = 9). Data were normalized based on corresponding total unphosphorylated protein then expressed in % relative to maximum phosphorylation value. * *p* < 0.05 compared with AGE-HSA and † < 0.05 compared with CML-HSA controls (0 min) using one-way ANOVA followed by Dunnett post-test. Results shows that both RAGE ligands increase ERK activity (Figures A and E) over 60 min of stimulation (AGE-HSA: 100±0% and CML-HSA: 82.6±8.6%) compared to basal phosphorylation (AGE-HSA: 62.7±5.4% and CML-HSA: 53.3±7.8%), whereas p38 activity (Figures B and F) was not significantly affected by the CML-HSA treatment. However, cells treated with AGE-HSA for 15 min (54.4±7.9%) showed a signal lower than basal p38 activity (86.1±4.7%). As for JNK phosphorylation (Figures C and G), both RAGE ligands decreased JNK signaling in A7r5. Indeed, when compared to basal JNK activity (91.7±4.5%), AGE-HSA decreased JNK phosphorylation after 15 min of stimulation (68.3±5.7%) and remained lower at 30 and 60 min (50.8±7.6% and 59.2±8.8%, respectively). In the case of CML-HSA, when compared to unstimulated controls (84.0±5.1), a significant reduction in JNK activity was also observed at 30 min (54.4±6.8%) and was maintained over 60 min of stimulation (59.6±8.8%).
(TIF)

S4 File RAGE signaling does not affect significantly AVP-induced cytoskeletal reorganisation. Figure A, Representative epifluorescence micrographs (40X) of Phalloidine/TexasRED stained cells showing actin organization. Figure B, Histogram of average TexasRED fluorescence corresponding to actin density, in A7r5 cells stimulated 24hours with 1mg/ml AGE-HSA, 1 mg/ml CML-HSA or PBS and challenged with 200 nM AVP. Each bar represents 9 independent experiments.
(TIF)

S1 Table Primer sequences used for qRT-PCR. *, indicates genes used as housekeeping for normalisation. F and R, indicate forward and reverse primer sequences, respectively.
(DOCX)
