# Supplementary figures and images for: Receptor for Advanced Glycation End-Products Signaling Interferes with the Vascular Smooth Muscle Cell Contractile Phenotype and Function
Source: PLoS One. 2015 Aug 6;10(8):e0128881. doi: 10.1371/journal.pone.0128881 (PMC4527751; doi:10.1371/journal.pone.0128881)

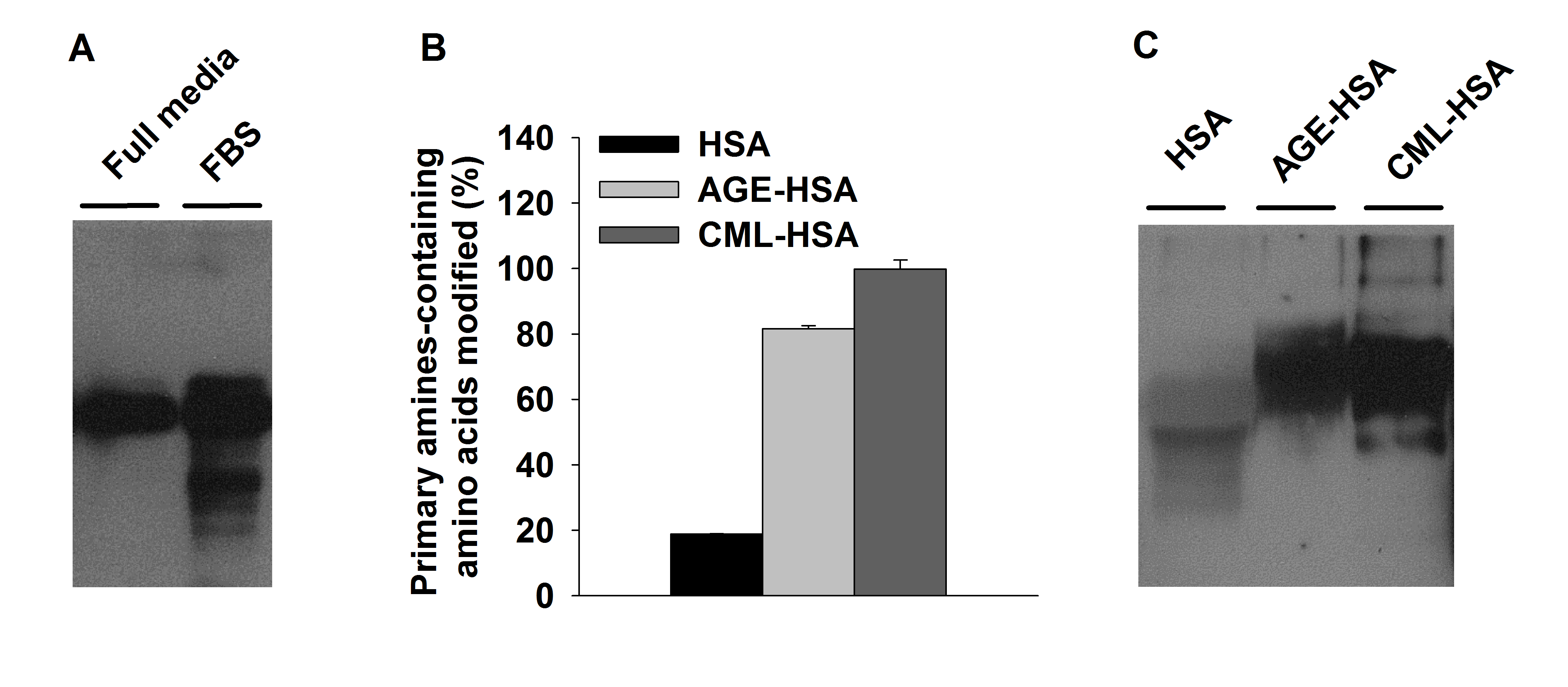

Supplement: S1 File — Human recombinant albumin was treated in order to produce AGE-HSA or CML-HSA. Figure A, A7r5 cell culture media containing AGE as revealed by immunobloting for CML adduct. These results confirm that glycation occurred in vitro and reveal that untreated HSA carries a certain degree of modification forbidding its use as an experimental control. Thus, PBS was used as a negative control in our study. Figure B, Degree of modification of amino acids containing primary amines using the TNBSA assay. The results show that untreated recombinant HSA has a degree of modification of 18.8±0.1% whereas HSA incubated with glucose (AGE-HSA) is extensively modified to 81.6±0.9%. Similarly, CML-HSA has more than 99.8±2.8% of its primary amines modified. Figure C, Presence of CML adducts in the different preparations by immunoblotting using a CML specific antibody. Results show that CML-HSA contains the CML adduct, which appears less prevalent in the AGE-HSA preparation and in the untreated HSA. (TIF) [file pone.0128881.s001.tif]

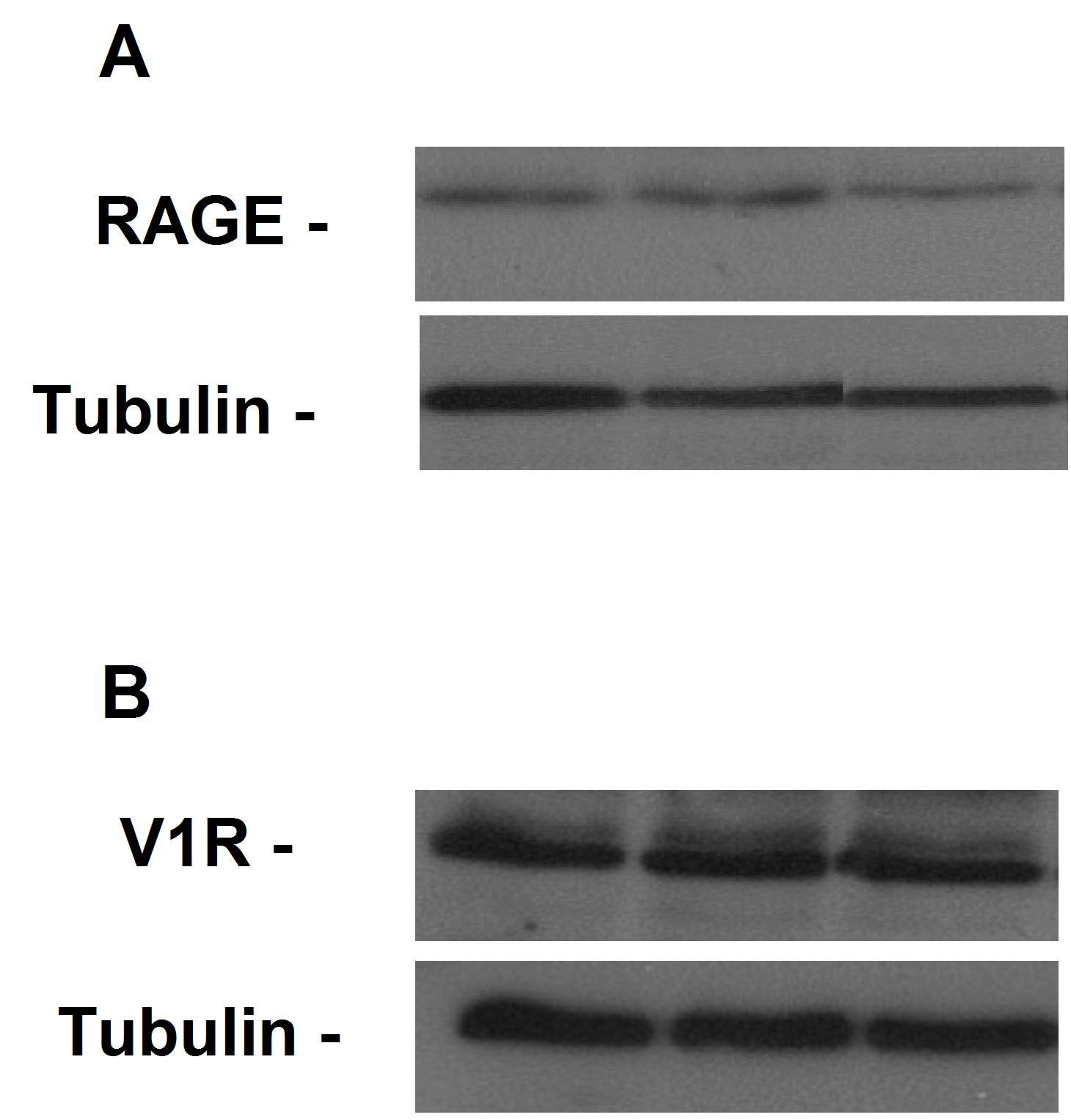

Supplement: S2 File — Immunoblots showing the expression of (Figure A) RAGE and (Figure B) V1R in A7r5. Tubulin was used as loading control. (TIF) [file pone.0128881.s002.tif]

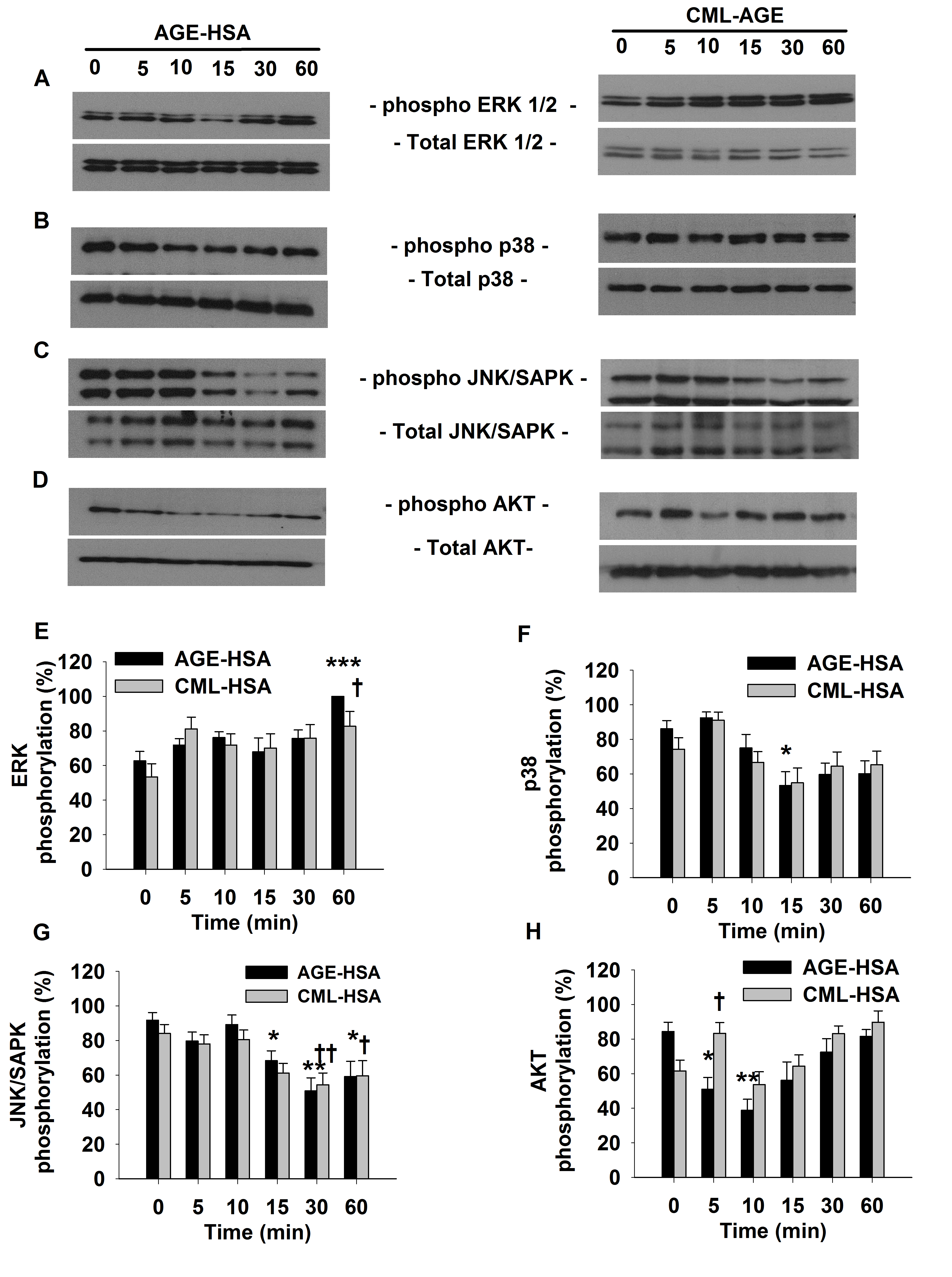

Supplement: S3 File — Immunoblots showing phosphorylated and total (Figure A) ERK 1/2, (Figure B) p38, (Figure C) JNK/SAPK or (Figure D) AKT proteins levels in A7r5 cells stimulated with (left panels) 1mg/ml AGE-HSA or (right panels) 1mg/ml CML-HSA at indicated times. Semi-quantitative analysis of (Figure E) ERK, (Figure F) p38, (Figure G) JNK/SAPK or (Figure H) AKT activation in A7r5 cells stimulated for indicated times with 1mg/ml AGE-HSA or 1 mg/ml CML-HSA (n = 9). Data were normalized based on corresponding total unphosphorylated protein then expressed in % relative to maximum phosphorylation value. * p < 0.05 compared with AGE-HSA and † < 0.05 compared with CML-HSA controls (0 min) using one-way ANOVA followed by Dunnett post-test. Results shows that both RAGE ligands increase ERK activity (Figures A and E) over 60 min of stimulation (AGE-HSA: 100±0% and CML-HSA: 82.6±8.6%) compared to basal phosphorylation (AGE-HSA: 62.7±5.4% and CML-HSA: 53.3±7.8%), whereas p38 activity (Figures B and F) was not significantly affected by the CML-HSA treatment. However, cells treated with AGE-HSA for 15 min (54.4±7.9%) showed a signal lower than basal p38 activity (86.1±4.7%). As for JNK phosphorylation (Figures C and G), both RAGE ligands decreased JNK signaling in A7r5. Indeed, when compared to basal JNK activity (91.7±4.5%), AGE-HSA decreased JNK phosphorylation after 15 min of stimulation (68.3±5.7%) and remained lower at 30 and 60 min (50.8±7.6% and 59.2±8.8%, respectively). In the case of CML-HSA, when compared to unstimulated controls (84.0±5.1), a significant reduction in JNK activity was also observed at 30 min (54.4±6.8%) and was maintained over 60 min of stimulation (59.6±8.8%). (TIF) [file pone.0128881.s003.tif]

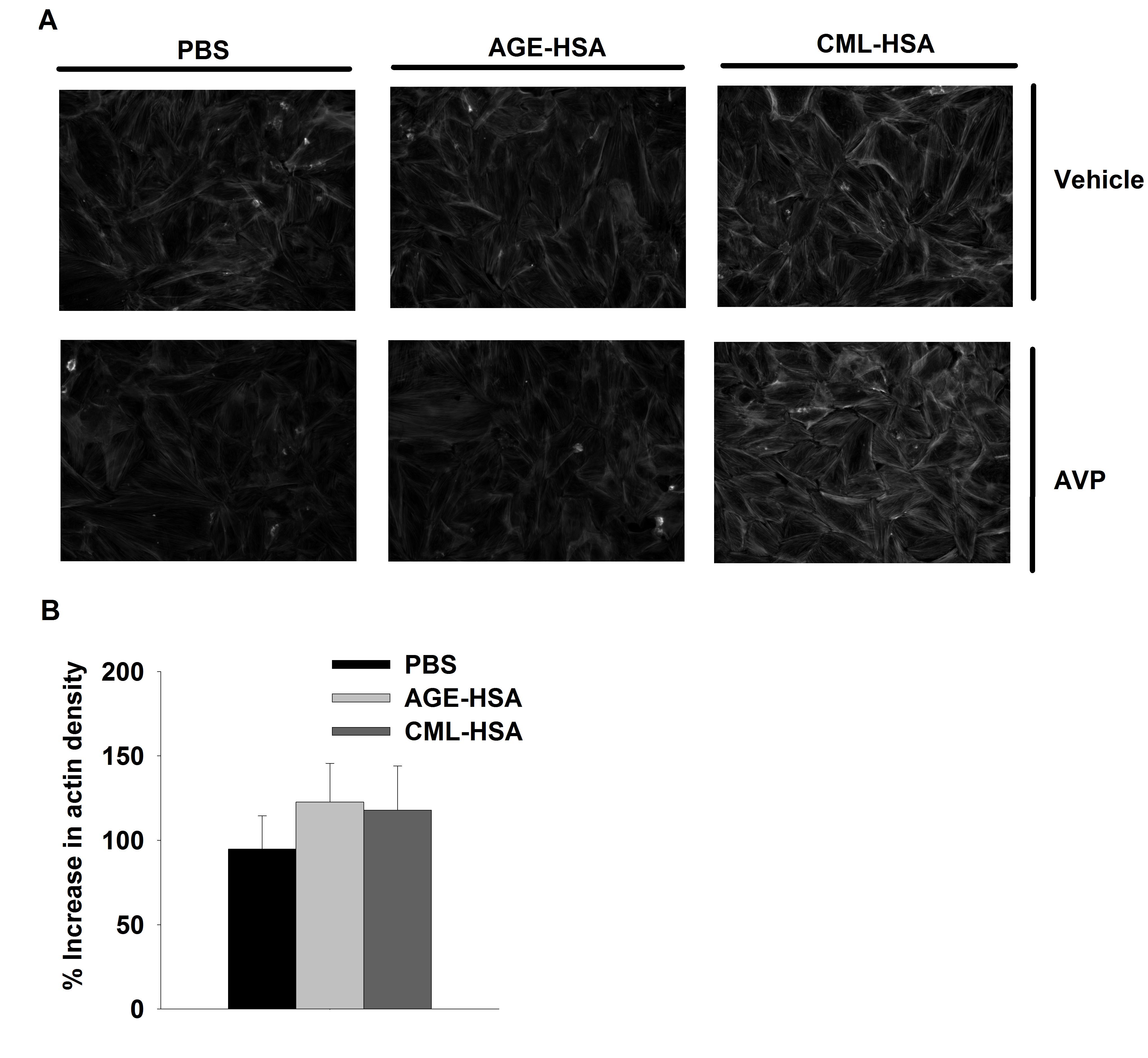

Supplement: S4 File — Figure A, Representative epifluorescence micrographs (40X) of Phalloidine/TexasRED stained cells showing actin organization. Figure B, Histogram of average TexasRED fluorescence corresponding to actin density, in A7r5 cells stimulated 24hours with 1mg/ml AGE-HSA, 1 mg/ml CML-HSA or PBS and challenged with 200 nM AVP. Each bar represents 9 independent experiments. (TIF) [file pone.0128881.s004.tif]
