# Supplementary material for: Receptor for Advanced Glycation End-Products Signaling Interferes with the Vascular Smooth Muscle Cell Contractile Phenotype and Function
Source: PLoS One. 2015 Aug 6;10(8):e0128881. doi: 10.1371/journal.pone.0128881 (PMC4527751; doi:10.1371/journal.pone.0128881)
Supplement: S1 Table — *, indicates genes used as housekeeping for normalisation. F and R, indicate forward and reverse primer sequences, respectively. (DOCX) [file pone.0128881.s005.docx]

| **Gene of interest** | **Sequences** |
| --- | --- |
| *****Pumilio RNA-binding family member 1 | **F:** CCG ACC AGA CCCT CCC TAT CCT |
|  | **R:** TGC TGA CTC AAT ACA AGT ACA TTG CCT C |
| *****Ribosomal protein L19 | **F:** TGT GGT AAA AAG AAG GTG TGG TTG GA |
|  | **R:** GGC CAA GGT GTT CTT CCG GC |
| *****Tubulin, beta 5 class I | **F:** GAC TCC GTT CGC TCA GGT CC |
|  | **R:**TTC CGC ACC ACA TCC AAG ACA GA |
| Smooth muscle alpha-actin | **F:** GGG CTG TTT TCC CAT CCA TCG T |
|  | **R:** GTG ATG ATG CCG TGT TCT ATC GGA |
| Smooth muscle myosin heavy chain | **F:** ACG GGA TGT GGT GCA GAA AGC T |
|  | **R:** CCC CCT GAC GAT GGG TCT TAT CC |
| Myocardin | **F:** AGA ACG ATA CAG CCT CCC AGC T |
|  | **R:** GGG TCT TTG GGC TTT TTG TGG C |
| Transgelin/SM22-alpha | **F:** TTC AAG CAG ATG GAA CAG GTG GC |
|  | **R:** ACT GCC CAA AGC CAT TAC AGT CC |
